# Oxidation as “The Stress of Life”

**DOI:** 10.18632/aging.100385

**Published:** 2011-09-21

**Authors:** Nikolay L. Malinin, Xiaoxia Z. West, Tatiana V. Byzova

**Affiliations:** Department of Molecular Cardiology, J.J. Jacobs Center for Thrombosis and Vascular Biology, Lerner Research Institute, Cleveland Clinic, Cleveland, OH 44195, USA

**Keywords:** lipid oxidation, oxidative stress, aging, Toll-like receptor 2, AMD, angiogenesis, atherosclerosis

## Abstract

Multiple biological consequences of oxidative stress are known to contribute to aging and aging-related pathologies. It was recently shown that (carboxyalkyl)pyrroles (CAPs), the end products of phospholipid oxidation serve as a novel class of endogenous ligands for Toll-like receptors (TLRs) and promote the process of angiogenesis. In this review, we discuss implications of these findings in the context of age-related pathologies, including tumorigenesis. Accumulation of oxidation products in tissues of aging organisms might create conditions for uncontrolled pathological angiogenesis as seen in patients with age related macular degeneration. CAPs and their receptors, TLRs might also promote the progression of atherosclerotic lesions. Importantly, besides their role in a number of pathologies, oxidative products of phospholipids contribute to tissue repair processes thereby antagonizing the destructive effects of oxidation.

Oxidative stress is considered to be a major detrimental factor limiting longevity [1], as originally postulated in the free radical theory of aging [2]. The oxidative stress leads to accrual of damaged/misfolded proteins [3], increased mutagenesis rate [4] and inflammation [5,6]. Ironically, due to its ability to accumulate over time (as it was seen in many neurodegenerative disorders) [3], oxidative damage also emerged as a consequence of longevity *per se*. The human life-span exceeds that of most mammalian species at least 4 times (median life span records across 900 mammalian species is ~16 years) [7]. Importantly, anti-oxidative stress adaptations are not subjects of evolutionary pressure at post-reproductive age, which further contributes to the buildup of oxidative damage in aging individuals. Yet, paradoxically, in short-lived *Caenorhabditis elegans,* oxidative stress might have beneficial effect on longevity [8,9] by connecting to the nutrition signaling pathways [10,11]. It was suggested that aging is driven by over-activation of signal-transduction pathways such as the nutrient-sensing pathway, while oxidative stress may be both one of its activators and effectors [12,13].

Both blood cells and the circulatory system are permanently exposed to high oxygen tension; therefore, the signs of oxidative damage are expected to be present in blood substantially earlier than in other tissues. However, red blood cells are renewed before the damage achieves critical levels [14], and similar a mechanism might be in place for endothelial cells. It is likely that the decrease in renewal of endothelial cells is a significant contributing factor to cardiovascular disorders of the aging blood vessels [15]. Extra-cellular matrix (ECM) is a less dynamic component of the vessel wall, therefore, the signs of aging such as mineral deposits [16], amyloid-beta peptide [17] and viral particles [18] accumulate in this particular compartment. Likewise, ECM might serve as a reservoir for oxidative stress products.

Outside of the circulation, wounds and tumors (often referred to as “never healing wounds” [19]) are known to be associated with a high degree of oxidative stress [20,21]. In these pathological settings, the presence of an oxidative stress sensor able to activate stress-responsive protective mechanism is crucial. However, elevated oxygen or reactive oxygen species (ROS) have relatively short life times, which is a disadvantage in terms of adaptation to oxidative stress. In contrast, generation of oxidation products - oxidative signature - is able to accumulate and propagate “stress” signals over prolonged period of time.

One example of this molecular signature of oxidation is extracellular matrix modifications resulting from the oxidative stress [22]. Carboxy-alkyl-pyrroles (CAPs) protein adducts have all the anticipated properties of the oxidative stress signature: they are generated as end-products of lipid oxidation and are able to form stable conjugates with ECM proteins. CAPs were originally isolated from retinas of patients with advanced age-related macular degeneration (AMD) [23], and later were also found in other organs with substantial oxidative stress [22].

CEP (carboxy ethyl pyrrole), the most abundant CAP in AMD, has pronounced angiogenic effect both *in vitro* and *in vivo* [22,24]. CAP-induced angiogenesis may be considered as a part of coordinated tissue responses aimed to adapt to damaging conditions. CAP-induced neo-vascularization might represent an additional line of defense against tissue damage (e.g. wound) beneficial for tissue supply with oxygen and nutrients as well as augment inflammatory response.

**Figure 1:**
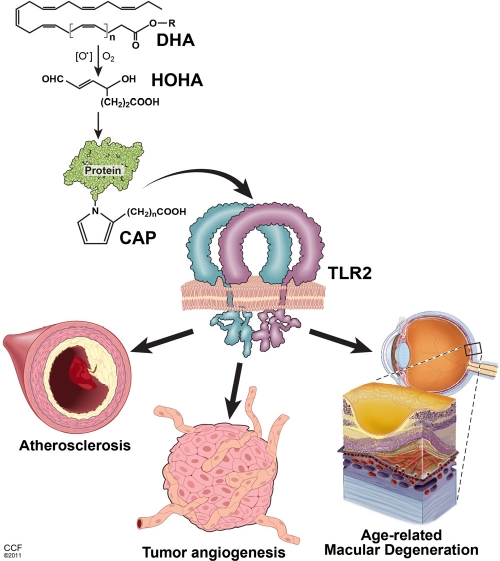
Key pathological effects of lipid oxidation products related to the aging Oxidation of poly-unsaturated fatty acids (exemplified by DHA - docosahexaenoic acid) leads to the generation of intermediate products (HOHA - 4-hydroxy-7-oxo hept-5-enoic acid) and, consequently, accumulation of carboxy-alkyl-pyrrole (CAP) protein adducts. CAPs stimulate TLR1/2 heterodimer on the surface of endothelial cells triggering angiogenesis. In turn, excessive CAP-induced angiogenesis contributes to the age-related macular degeneration and tumor progression. Accumulation of HOHA and CAPs in blood vessel walls may also constitute a substantial component of pathophysiological changes during atherosclerosis.

In our recent report [22] we identified Toll-like receptor 2 (TLR-2) as a main receptor and sensor for CAP protein adducts. The other likely candidates for this function - scavenger receptors, including CD36 [25,26], were not required for CAPs sensing. Originally described as innate immunity receptors [27], TLRs, in addition to the ligands of pathogen-associated molecular patterns (PAMP), have been shown to recognize a number of endogenous ligands associated with non-infectious (sterile) inflammation [28]. As a result, the concept of Damage Associated Molecular Patterns (DAMPs) which included PAMPs as a particular case, has evolved [28,29]. Thus, identification of CAPs as endogenous ligands for TLRs was not entirely surprising. However, this family of ligands was unique as it represented a “molecular signature” of the oxidative stress. Moreover, TLR2/CAPs axis exemplifies a novel oxidative-damage sensing mechanism aimed to promote vascularization. In an independent study, it was reported that TLR2/6 complex was involved in angiogenic response upon stimulation with bacterial lipoprotein [30].

CAPs-induced angiogenesis is a peculiar example of coordinated response by various tissues to the plethora of TLR ligands *in vivo.* Whereas PAMPs serve as potent modulators of immune system, CAPs promote angiogenesis via activation of endothelial cells. Whether CAPs are also able to affect immune system will be the subject of future studies. Noteworthy, robust CAPs-dependent angiogenesis was originally described in the eye [23] - an organ of immune privilege, which separates effects of TLR ligands on endothelial cells from that of invading leukocytes [31]. Likewise, angiogenesis was diminished in tumors grown in TLR2-null hosts, independently of TLR2 expression on bone marrow derived cells [22]. Experiments using isolated endothelial cells and *in vivo* wound closure assays on wild type and TLR2 knockout chimera animals confirmed the conclusion that effects of CEP was mediated by endothelial cells [22].

Another intriguing feature of CAP-induced angiogenesis is the independence from VEGF receptor tyrosine kinase activity [22]. Therefore, TLR2 signaling represents a unique pathway contributing to tumor vascularization, thereby providing an explanation for the unexpectedly low efficacy of VEGFR inhibitors in certain cancers [32]. However, under physiological conditions, it appears that both TLR2 and VEGFRs are aimed to promote formation of blood vessels in a well coordinated manner.

The pro-angiogenic function of TLR2 highlights the importance of both transcription-dependent and independent signaling pathways induced by Toll-like receptors. TLR2 has been shown to modulate levels of HIF-1alpha expression [33], which is also modulated by genotoxic streess [34] and aerobic glycolysis [35], providing additional node to the signaling network contributing to cancerogenesis [36]. Transcription-independent branch involves CEP-induced activation of Rac1 small G-protein, which modulates cell adhesion and migration, and known to be activated downstream of TLR2 [37]. Furthermore, Rac1 mediated ROS production could contribute both to genotoxic response and lipid oxidation, creating self-perpetuating cycle of oxidative damage [38].

Interestingly, different Toll-like receptors might have opposing effects on angiogenesis. Double stranded RNA, a known ligand for TLR3, has been reported to block angiogenesis in AMD [39]. The differential outcomes of TLR2 vs TLR3 stimulation might be result of interference between TLR2 and TLR3 -induced signaling pathways [40]. In the context of a whole tissue, the signaling induced by same TLR expressed on different cells combined with the interplay between different TLRs might allow coordination of tissue repair and immune response.

TLR2-dependent angiogenesis may be considered as a tissue adaptation to oxidative stress, most commonly found in wound [20]. Indeed, CEP is abundant in wounded tissue, and anti-CEP blocking antibodies decreased neo-vascularization and wound recovery, apparently without compromising immune response at the site of damage [22]. Both tissue remodeling and immune response induced by Toll like receptor 2 can be considered as manifestations of a general damage/stress response at the tissue level. In a far-fetched analogy, an intriguing example of systemic TLR dependent stress adaptation may be a radioprotective effect of TLR5 stimulation [41].

However, as it is often the case in complex systems, too much of a good thing is bad, and an aberrant TLR-dependent signaling is known to contribute to a number of pathologies ranging from allergies [42] to cancer [43,44]. While contributing to wound healing, CAPs seem to accumulate with age, most notably in blood vessels walls [22]. The accumulated CAPs might be involved in chronic inflammation linked to cardiovascular disorders. A significant example of this is the progression of atherosclerosis, which has been demonstrated to be a TLR2 dependent process [45]. Thus, elevated CAPs content could be an age-related risk factor associated with the disease.

In summary, oxidative stress creates a relatively stable molecular pattern in the form of carboxy-alkyl pyrroles-modified proteins. Accumulation of CAPs results in activation of TLR2 receptor complexes on endothelial cells, providing a stress-responsive mechanism for tissue remodeling through induction of angiogenesis. Thus, the concept of stress adaptation, put forth by Hans Selye more than 75 years ago [46], has received yet another mechanistic explanation.
